# Crude Oil and Its Burnt Residues Induce Metamorphosis in Marine Invertebrates

**DOI:** 10.1021/acs.est.3c05194

**Published:** 2023-11-14

**Authors:** Rodrigo Almeda, Sinja Rist, Anette M. Christensen, Eleftheria Antoniou, Constantine Parinos, Mikael Olsson, Craig M. Young

**Affiliations:** † EOMAR-ECOAQUA, University of Las Palmas de Gran Canaria, 35017 Tafira Baja, Las Palmas, Spain; ‡ National Institute of Aquatic Resources, Technical University of Denmark, 2800 Kongens Lyngby ,Denmark; § Oregon Institute of Marine Biology, University of Oregon, Charleston, Oregon 97420,United States; ∥ School of Chemical and Environmental Engineering, Technical University of Crete, 73100 Chania, Greece; ⊥ School of Mineral Resources Engineering, Technical University of Crete, 73100 Chania, Greece; # Hellenic Centre for Marine Research (HCMR), Institute of Oceanography, 19013 Anavyssos, Attiki, Greece; ▲ DTU Sustain, Technical University of Denmark, 2800 Kongens Lyngby, Denmark

**Keywords:** metamorphosis, crude oil, planktonic larvae, pollution, benthic recruitment

## Abstract

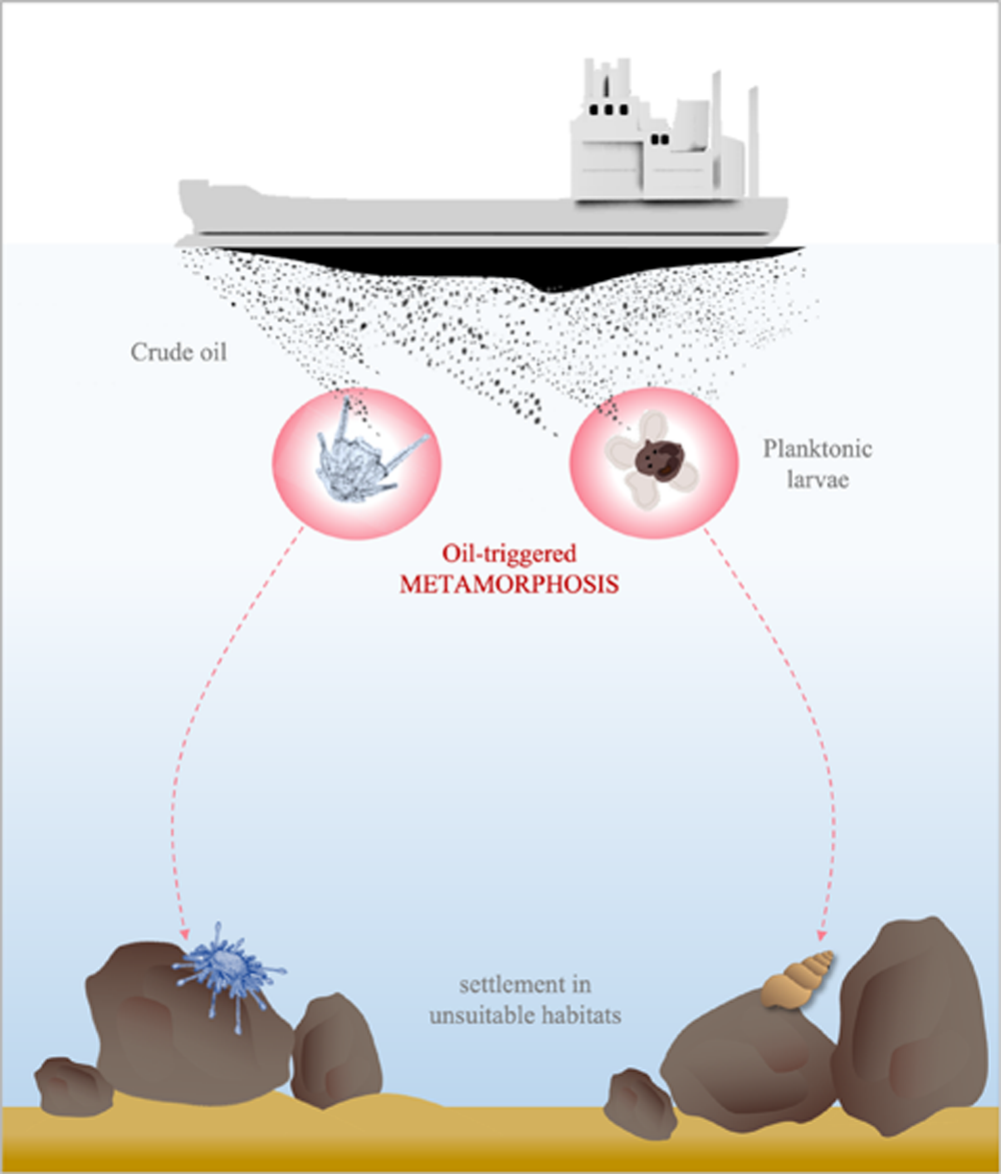

Metamorphosis is a critical process in the life cycle of most marine benthic invertebrates, determining their transition from plankton to benthos. It affects dispersal and settlement and therefore decisively influences the dynamics of marine invertebrate populations. An extended period of metamorphic competence is an adaptive feature of numerous invertebrate species that increases the likelihood of finding a habitat suitable for settlement and survival. We found that crude oil and residues of burnt oil rapidly induce metamorphosis in two different marine invertebrate larvae, a previously unknown sublethal effect of oil pollution. When exposed to environmentally realistic oil concentrations, up to 84% of tested echinoderm larvae responded by undergoing metamorphosis. Similarly, up to 87% of gastropod larvae metamorphosed in response to burnt oil residues. This study demonstrates that crude oil and its burned residues can act as metamorphic inducers in marine planktonic larvae, short-circuiting adaptive metamorphic delay. Future studies on molecular pathways and oil-bacteria-metamorphosis interactions are needed to fully understand the direct or indirect mechanisms of oil-induced metamorphosis in marine invertebrates. With 90% of chronic oiling occurring in coastal areas, this previously undescribed impact of crude oil on planktonic larvae may have global implications for marine invertebrate populations and biodiversity.

## Abstract 2

This study demonstrates for the first time that crude oil induces metamorphosis in planktonic larvae, which can potentially have a profound impact on marine invertebrate populations and biodiversity.

## 1 Introduction

The phenomenon of metamorphosis has fascinated humanity from the earliest ancient myths to the first descriptions of life cycles in butterflies.^1^ In contrast to humans and other terrestrial vertebrates with direct development, most marine animals have indirect development with metamorphosis occurring at some stage during their life history, most often at the transition from planktonic larvae to benthic juveniles in species with biphasic life cycles.^2^ These larvae, known as meroplankton, may differ from adults in form, size, feeding behavior, habitat, locomotion, and dispersal capability.^3−5^ A planktonic larval stage provides a means to disperse and colonize new suitable habitats, reducing the risk of extinction after local disturbances and enabling connectivity and genetic flow among metapopulations.^6−9^

Metamorphosis, which terminates the larval stage, is recognized as a critical life process in marine invertebrates affecting dispersal, settlement, recruitment success, population dynamics,^6,10−12^ and ultimately biodiversity in marine environments.^13^ When larvae complete their development and have the capacity to undergo metamorphosis, they are referred to as “competent larvae”.^14^ In many marine benthic invertebrates, metamorphosis is induced by external factors detected by the competent larvae; thus, larvae do not settle randomly on the seafloor but rather select sites with appropriate resources or habitats that are needed for the survival of juveniles or adults.^15−20^ Metamorphosis in invertebrate larvae is sometimes postponed for long periods in the absence of specific chemical cues and/or substrata that indicate favorable conditions for settlement.^14,15^ Some planktonic larvae have adaptions for long-distance dispersal across the oceans (“teleplanic larvae”),^8^ and their larval duration can be extraordinarily long, years in some cases,^20^ in the absence of specific environmental cues. External cues for metamorphosis may include chemicals from needed vegetation or prey, microbial signaling molecules from biofilms, and conspecific exudates.^10,21−27^ In some invertebrate species, adverse conditions such as food limitation^28^ and thermal stress^29,30^ may trigger metamorphosis in larvae, yet the presence of potential competitors may stimulate delayed metamorphosis.^31,32^

Delay of metamorphosis (in the sense that competent larvae can stay in that developmental stage for extended periods of time in the absence of a specific metamorphic trigger or cue) is generally considered an adaptive strategy for assuring settlement in habitats suitable for subsequent survival, growth, and reproduction. In an extensive review, Pechenik^33^ documented a facultative delay of metamorphosis in 75 species of 14 phyla, including mollusks and echinoderms. Although delay often involves trade-offs (e.g., settlement size may be smaller because of energy depletion in larvae), it may also improve the likelihood of postsettlement survival. An example of the latter occurs in the sand dollar *Dendraster excentricus*, one of the species we studied. Highsmith^34^ demonstrated that the competent larvae of this species delay settlement until encountering peptides in sand occupied by conspecific adults. Reworking sediment by adult sand dollars prevents the establishment of an abundant crustacean that is a major predator on larval and juvenile sand dollars. Thus, gregarious settlement following metamorphic delay is an adaptive behavior that likely ensures successful recruitment.

Pollution is a major anthropogenic stressor in coastal waters. Studies on the effects of pollution on meroplanktonic larvae typically have focused on the survival and growth of early development stages in a few model species.^35−37^ In contrast, we know little about the influence of pollution on larval metamorphosis and settlement. Oil pollution in marine environments, both acute and chronic oiling, is a major global environmental problem.^38^ Crude oil is the largest primary energy source in the world^39^ and is mainly transported over maritime shipping routes and by underwater pipelines.^40,41^ Despite the efforts of the oil industry to reduce the number of oil spills, accidental oil spills seem to be inevitable. The Deep-Water Horizon oil rig explosion in the Gulf of Mexico (2010), considered “potentially the worst environmental disaster in American history”, (Obama 2010) and the spills from broken pipelines in Borneo (2018) and Thailand (2022) are just some examples of catastrophic oil spills. Coastal waters are also exposed to chronic oil pollution from anthropogenic sources. Dong et al. (2022) found that globally 90% of oil slicks occur within 160 km of the coasts.^38^ Coastlines concentrate a large number and biodiversity of marine benthic invertebrates. The consequences of accidental and global chronic oiling on the metamorphosis of invertebrates are unknown despite the relevance of this biological process in the dynamics of marine coastal ecosystems.

In this study, we present the first evidence that metamorphosis in marine invertebrates can be induced by exposure to crude oil. We investigated the effects of oil on the metamorphosis of invertebrate larvae from two different taxa: gastropods and echinoids. In veliger larvae of gastropods, we demonstrated, surprisingly, that burnt oil compounds induce metamorphosis rather than disrupting it. A similar result was obtained in the competent echinopluteus larvae of sand dollars: unburnt crude oil accelerated metamorphosis at all concentrations, shortening larval life, with potentially important consequences for settlement success and survival.

## 2 Materials and Methods

### 2.1 Experiments with Gastropod Larvae

#### 2.1.1 Sampling of Larvae

The gastropod larvae used in the experiments (*Rissoa* sp.) were obtained from zooplankton samples collected from coastal surface waters located 5 nautical miles north of Heraklion, Crete (35° 24.957 N, 25° 14.441 E) by horizontally trawling a WP-2 plankton net (45 μm mesh) in May 2018. The contents of the net cod-end were transferred to cool boxes, diluted with in situ seawater, and transported to the laboratory within 2 h of collection. There, the larvae were identified under a dissecting microscope, sorted with a glass pipet, and placed in 2 L beakers with 20 μm filtered seawater (FSW).

#### 2.1.2 Experimental Design

**Figure 1:**
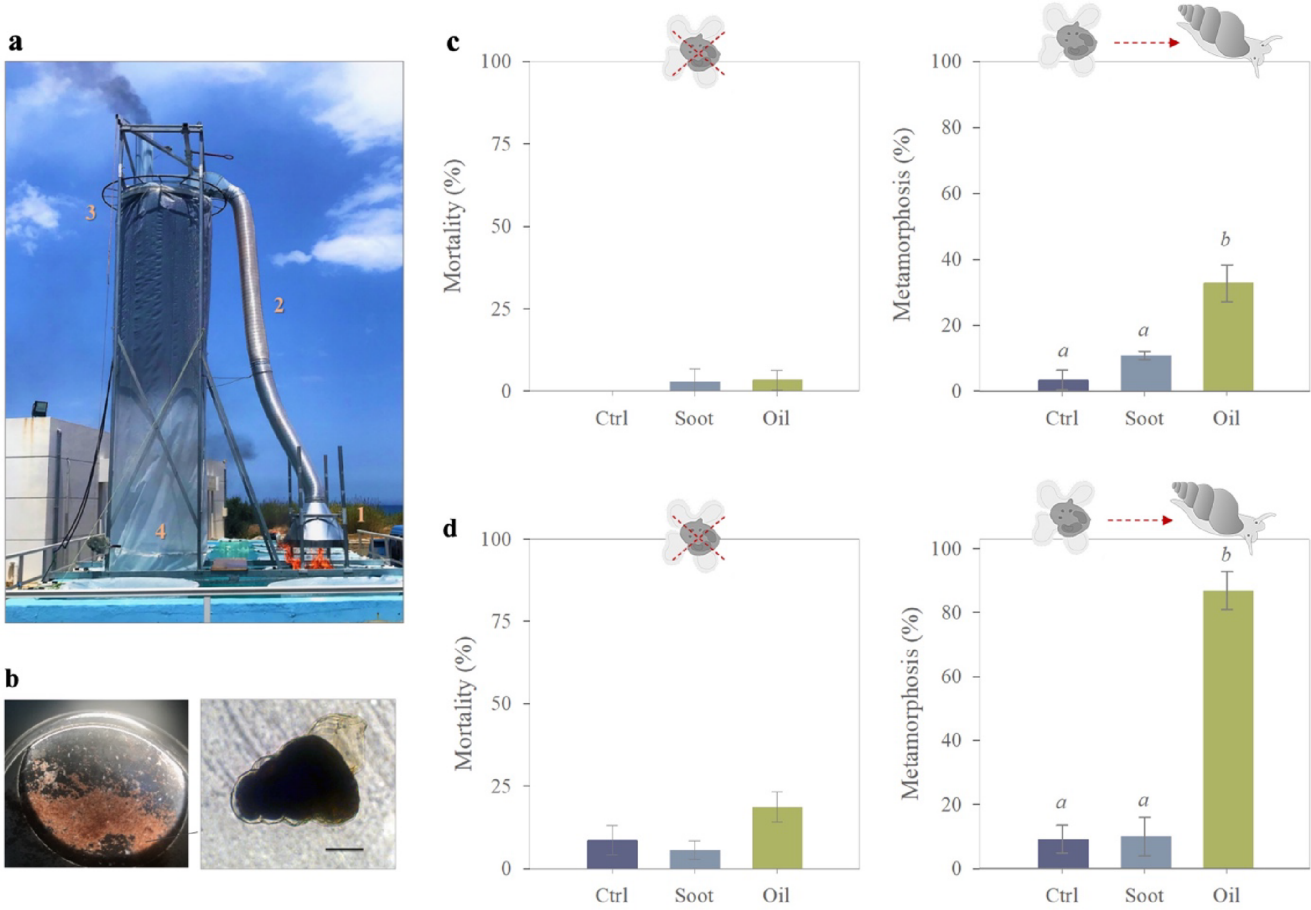
Survival and metamorphosis of gastropod (*Rissoa* sp.) veliger larvae after 3 days of exposure to oil burning byproducts (soot and burnt oil). (a) Experimental setup used to obtain soot and residues of burnt oil in the mesocosms (1: crude oil burning, 2: soot emissions collected, 3: rain simulation, 4: deposition of soot on the mesocosm). (b) Plankton net sample with high concentration of gastropod larvae (left) and individual gastropod larva (right), scale bar = 100 μm. (c) Effect of oil burning byproducts 1 day after burning on survival and metamorphosis of gastropod veliger larvae. (d) Effect of oil burning byproducts 6 days after burning on survival and metamorphosis of gastropod veliger larvae (Ctrl= control without pollutants, Soot, Oil= burnt oil). Lowercase italic letters (*a*, *b*) indicated different statistical groups (*p* < 0.05).

The experiments with gastropod larvae were a side study of a joint mesocosm experiment conducted at the Hellenic Centre for Marine Research in Crete (Greece) to evaluate the impacts of in situ oil burning on marine plankton.^42^ The mesocosms consisted of transparent food-grade polyethylene bags mounted on circular metal frames attached to a land-based open pool (350 m^3^, 5 m deep) with a continuous flow of in situ seawater. The mesocosms were filled with 3.5 m^3^ of seawater collected at 1 m depth from a Cretan Sea coastal station (0.2 miles off the North coast of Crete) using a rotary submersible pump in May 2018. The surface seawater was transported to land in 100 L acid-washed plastic containers within ∼3 h and distributed sequentially into the mesocosms by gravity siphoning using plastic tubes connected to a flowmeter. To simulate an oil spill burning and wet deposition of soot, a structure was designed and built to obtain and separate burnt oil residues and soot after crude oil burning (Figure 1A). Briefly, 2 L of Iranian crude oil (0.57 mL L^–1^) were poured inside of a metal ring placed in the middle of a mesocosm. The oil was then ignited and burned, and the soot emissions were collected in a metal tube. The soot was finally deposited in the designated mesocosm by rain simulation. We repeated the procedure to obtain the soot and burnt residues for the different mesocosm replicates. The experimental setup involved nine mesocosms and the following treatments: (1) residues of burnt oil (triplicates B1–B3), (2) soot (triplicates S1–S3), and control without pollutants (triplicates C1–C3), and exposure lasted for 26 days.

#### 2.1.3 Exposure Experiments with Gastropod Larvae and Chemical Analyses

Experiments with gastropod larvae were conducted in glass bottles with water collected from the mesocosm treatments described above. Three exposure experiments of 72 h were carried out; in the first two experiments, gastropod larvae were exposed to water collected at 1 m depth from the mesocosms 1 and 6 days after oil burning. In the third experiment, gastropod larvae were exposed to residues of burnt oil from the B1–B3 mesocosms, 10 days after burning, with and without the addition of food ad libitum (*Isochrysis galbana*, exposure concentration: 50,000 cells mL^–1^) to evaluate if food limitation in the water from the oil mesocosms could cause metamorphosis.

Before starting the experiment, gastropod larvae were grouped in Petri dishes with 0.2 μm FSW. To start a microcosm experiment, seawater from each mesocosm was siphoned directly into the experimental glass bottles (1 L) and the bottles were immediately transported to the laboratory to add the larvae (20–25 larvae per bottle). Finally, the bottles were hung in a “floating wheel” at 0.5 m depth in the open pool where the mesocosms were established to ensure similar light and temperature exposure conditions.^43^ After incubation (72 h), the bottle contents were filtered through a 60 μm mesh sieve to collect the larvae in the laboratory. We assessed survival (beating of velar cilia for larvae, movement in juveniles) and metamorphic success (% of juveniles in the total number of living individuals as indicated by loss of the velum^44^ using a stereomicroscope).

The concentrations of 16 parent polycyclic aromatic hydrocarbon (PAH) compounds were determined in the water collected from the mesocosms and used as exposure media in the gastropod larvae tests (Figure S1, Supporting Information). Briefly, 2.5 L of water were collected in amber glass bottles, and after the addition of perdeuterated internal standards, the samples were extracted with 50 mL ultrapure hexane (SupraSolv., Merck) (*n*-C_6_).^45,46^ The *n*-C_6_ extract was filtered through activated Na_2_SO_4_ to absorb the moisture and concentrated using a rotary evaporator to remove the *n*-C_6_ solvent, then transferred in 100 μL glass inserts, using ultrapure dichloromethane (SupraSolv., Merck) (DCM), and analyzed with gas chromatography–mass spectrometry (GC–MS). The GC–MS analysis was performed using an Agilent GC–MS HP 7890/5975C system, with an Agilent HP-5 5% phenyl methyl siloxane column (30 cm × 250 μm × 0.25 μm) (Agilent Technologies). The analysis was carried out in single-ion detection (SIM) mode. The samples were injected diluted in 100 μL ultrapure DCM and spiked with the IS to 100 ppb concentration. The aromatic hydrocarbon components were quantified against the internal standard using an assumed response factor of 1 (see Antoniou et al. (2022)^47^ for further details regarding the instrumental analysis parameters). The precision of the analytical method, evaluated in terms of the repeatability of the experimental results (*n* = 8; in spiked samples) and expressed in terms of relative standard deviation, was ranged from 1.6 to 4.2% for individual PAHs. Procedural blanks were found to be free of any interference.

#### 2.1.4 Data Analysis

Data were analyzed with IBM SPSS Statistics 25.0. The assumptions of normality and homogeneity of variances were tested with the Shapiro–Wilk test and the Levene test, respectively. When the data followed the assumptions for parametric tests, a one-way analysis of variances (ANOVA) and Tukey’s HSD post hoc test were used to assess statistically significant differences among treatments (*p* < 0.05). When the data did not follow these assumptions, we used nonparametric Kruskal–Wallis tests with pairwise comparisons to determine significant differences between treatments (*p* < 0.05).

### 2.2 Experiment with Echinoderm Larvae

#### 2.2.1 Adult Sampling and Larval Culturing

Adult sand dollars (*Dendraster excentricus*) were collected by dredging outside of Coos Bay, Oregon (43° 24′21 N, 124° 19′43 W) at a depth of 12–20 m. They were kept in the laboratory in a flow-through seawater system with sand at the Oregon Institute of Marine Biology (OIMB), USA. Spawning was induced by injecting 1 mL of a 0.55 M potassium chloride (KCl) solution into the coelom by inserting a needle in the peristomial membrane near the mouth. Released gametes were collected in beakers with FSW, and gamete quality was checked with a stereomicroscope. A small amount of sperm was added to a diluted egg suspension for fertilization. Successful fertilization was confirmed by observing the development of the fertilization envelopes around most eggs. The suspension was divided into four glass bowls, diluted with FSW, and kept in a flow-through sea table at 13 °C. One day post fertilization (dpf), cellular debris and unfertilized eggs were carefully removed from the bowls with glass Pasteur pipettes. Since the embryos in all four bowls were developing well, they were mixed 2 dpf and divided into two 1.5 L glass jars. The number of individuals was adjusted to 2 mL^–1^. The jars were placed on the sea table with constant, gentle stirring. At 5 dpf, we started feeding the cultures with a mixture of *Rhodomonas salina* and *Dunaliella salina*. From then on, the cultures were fed twice per week, following a water change, in which approximately 80% of the water in the culture jars was removed by reverse filtration with a 110 μm sieve. This was replaced with fresh FSW. The larval development was checked regularly with a microscope, and the experiment was performed when competent larvae were observed at 11 weeks old.

#### 2.2.2 Crude Oil Preparation and Chemical Analysis

The crude oil used in this experiment was a Light Louisiana Sweet oil. A suspension of oil droplets was prepared by adding oil to seawater under high-speed magnetic stirring. The detailed method is described in Almeda et al. (2021).^48^ This procedure results in oil droplets with a mean diameter of 8 μm (95% of droplets between 1 and 20 μm), which has previously been analyzed with an imaging particle analysis system (FlowSight).^49^ The concentration and composition of PAHs in the crude oil suspensions were measured by using solid-phase extraction (SPE) and GC–MS. Briefly, a 100 mL sample was extracted using ENVI-18 SPE cartridges (6 mL, 1 g, Supelco). The columns were conditioned by 2 × 6 mL toluene:methanol 9:1 (v/v) followed by 6 mL methanol and 6 mL Milli-Q grade water. The sample was loaded at 10 mL min^–1^, and the columns were vacuum-dried for 1 h after loading. The PAHs were eluted using 2 mL of toluene:methanol 9:1 (v/v). For analysis, chromatographic separation was achieved on a Trace 1300 gas chromatograph (Thermo Scientific) equipped with a 60 m × 0.25 mm i.d × 0.25 μm film thickness HP-5 ms column (Agilent Technologies). A 1 μL sample was injected in splitless mode with the sample inlet held at 300 °C. The oven was programmed to 70 °C, then 20 °C min^–1^ to 300 °C, and then 50 °C min^–1^ to 325 °C held for 10 min. Helium was used as the carrier gas with a 1 mL min^–1^ constant flow. Detection was achieved on a Thermo Fischer ISQ-7000 mass-selective detector operated in SIM mode with the MS source at 230 °C and the quadrupole at 150 °C.

#### 2.2.3 Exposure Experiment with Competent Larvae

Competent larvae, which are recognizable by a clearly visible well-developed juvenile rudiment, were sorted from the culture and kept in a beaker of seawater until the start of exposure. The experiment had a full two-factorial design with the first factor being crude oil concentration and the second factor being temperature. Crude oil concentration had six levels (0, 5, 10, 25, 50, and 100 μL L^–1^), and temperature had two levels (ambient temperature at 13 °C and increased temperature at 18 °C). The increased temperature was chosen to reflect a marine heat wave, such as the eastern Pacific experienced in 2014–2016.^50^ We had triplicates of all 12 treatments.

Exposures were conducted in 20 mL glass scintillation vials with aluminum foil under the lid to prevent contact between the water and the plastic lid. All glassware was acid-washed and subsequently rinsed with reverse osmosis (RO) water prior to the experiment. Exposure vials were prepared as follows: Vials were almost filled with FSW and the desired volumes of the crude oil suspension were added using a micropipet with a glass tip. Then, vials were vigorously shaken before 10 presorted larvae were added using glass Pasteur pipettes. Lastly, vials were topped up with FSW to reach a total volume of 20 mL. All vials were wrapped in aluminum foil to exclude any influence of light. The vials for all ambient temperature treatments were placed in a sea table with flow-through of seawater from the bay close to the OIMB. The vials for the heat wave treatments were placed in a water bath in a temperature-controlled room. Every 12 h, the vials were inverted three times and water temperatures were recorded. At the end of exposure after 72 h, the content of each vial were poured into a small glass bowl. All larvae and juveniles were transferred to watch glasses for easier observation. Each individual was checked for the state of metamorphosis, signs of malformations, and survival.

#### 2.2.4 Data Analysis

All statistical analyses were done with the software R (version 3.6.3).^51^ For each measured response variable (i.e., percent metamorphosis, percent mortality, and percent malformations), a two-factorial ANOVA was conducted to check for main effects of the two independent variables (crude oil concentration and temperature) as well as their interaction. When no interaction was found, individual one-way ANOVAs were performed for each temperature. In the case of a significant finding, a post hoc test (Tukey’s HSD) was conducted. The assumption of normality of the residuals was tested with the Shapiro–Wilk W test, and the homogeneity of variances was tested with the Fligner-Killeen test. For mortality, we additionally calculated the relative median lethal concentration LC_50_ (i.e., the concentration at which 50% of individuals die) with the drc package.

## 3 Results

### 3.1 Effects of Burnt Oil and Soot on Survival and Metamorphosis of Gastropod Larvae

Mortality of larvae in the first two exposure experiments was very low in all treatments (0–8%) except for the burnt oil treatment in the second experiment, where mortality increased to 19% (Figure 1c,d). However, there were no statistically significant differences in mortality among treatments in the first (Kruskal–Wallis H test: χ2(2) = 2.047, *p* = 0.300) or second experiment (Kruskal–Wallis H test: χ2(2) = 5.793, *p* = 0.055). In the first experiment (1 day after oil burning), 33% of larvae exposed to burnt oil compounds had metamorphosed after 72 h, which was 10 times higher than in the control (ANOVA: *F*(2,5) = 42.501, *p* = 0.001; Tukey’s HSD: *p* < 0.05) (Figure 1c). In the second experiment (6 days after oil burning), the fraction of metamorphosed larvae in the oil treatment was nine times higher than in the control (ANOVA: F(2,6) = 198.379, *p* < 0.0001; Tukey’s HSD: *p* < 0.0001) and reached 87%. There was no statistically significant difference in metamorphosis between the control and the soot treatment in the two first experiments (ANOVA: *p* > 0.05) (Figure 1c,d).

**Figure 2:**
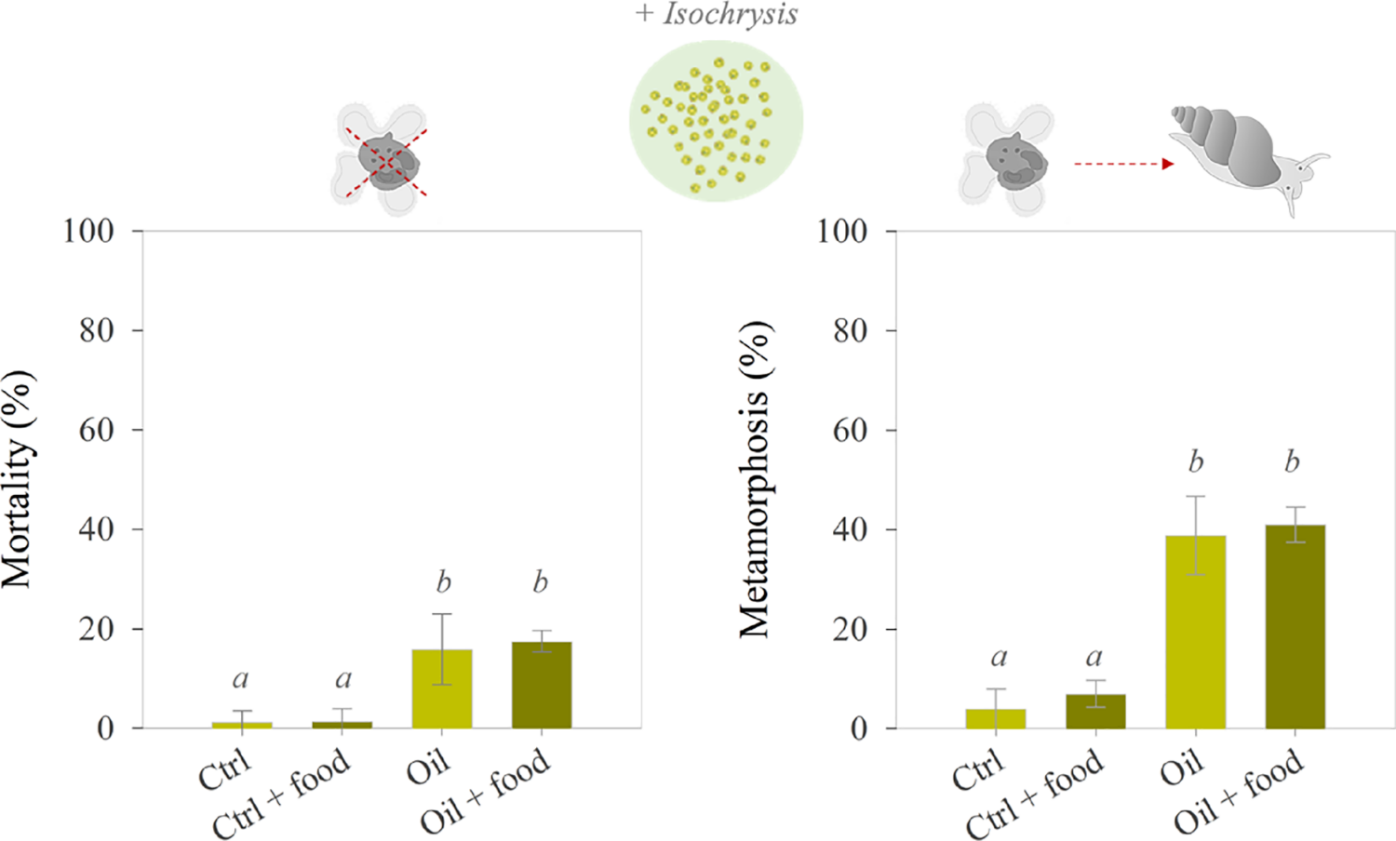
Effect of burnt oil on survival (left panel) and metamorphosis (right panel) of gastropod (*Rissoa* sp.) veliger larvae after 3 days of exposure to water collected from the mesocosms (10 days after burning) with or without the addition of food ad libitum (*Isochrysis galbana*, 50000 cells mL^–1^) (Ctrl= control without pollutants, Oil= burnt oil). Lowercase italic letters (*a*, *b*) indicated different statistical groups (*p* < 0.05).

In the third experiment (10 days after oil burning), to test the potential effect of food availability on metamorphosis, we found larval mortality of 16–18% in the oil treatment, which was significantly higher than the mortality in the control (Kruskal–Wallis H test: χ2(3) = 8.781, *p* = 0.032). Both for the oil and control treatments, there were no differences with or without added food (Figure 2). Similar to the two first experiments, metamorphosis increased up to 10 times when larvae were exposed to burnt oil compounds compared to the control (ANOVA: *F*(3,8) = 48.345, *p* <.0001) (Figure 2). Again, the addition of food did not affect metamorphosis (ANOVA: *p* > 0.05) (Figure 2).

The concentration and composition of PAHs detected in the exposure solutions (control, soot, and burnt oil residue) used in the gastropod larva experiment can be found in the Supporting Information (Figure S1). The highest concentration of PAHs was found in the water from the burnt oil treatment. The concentration of PAHs decreased with time after oil burning (1 > 6 > 10 d). Naphthalene, dibenzothiophene, phenanthrene, dibenzo[*a,h*]anthracene, benzo[*a*]pyrene, and fluorene were the most abundant PAHs in the burnt oil exposure solution (Figure S1).

### 3.2 Effects of Crude Oil on Sand Dollar Larvae

**Figure 3:**
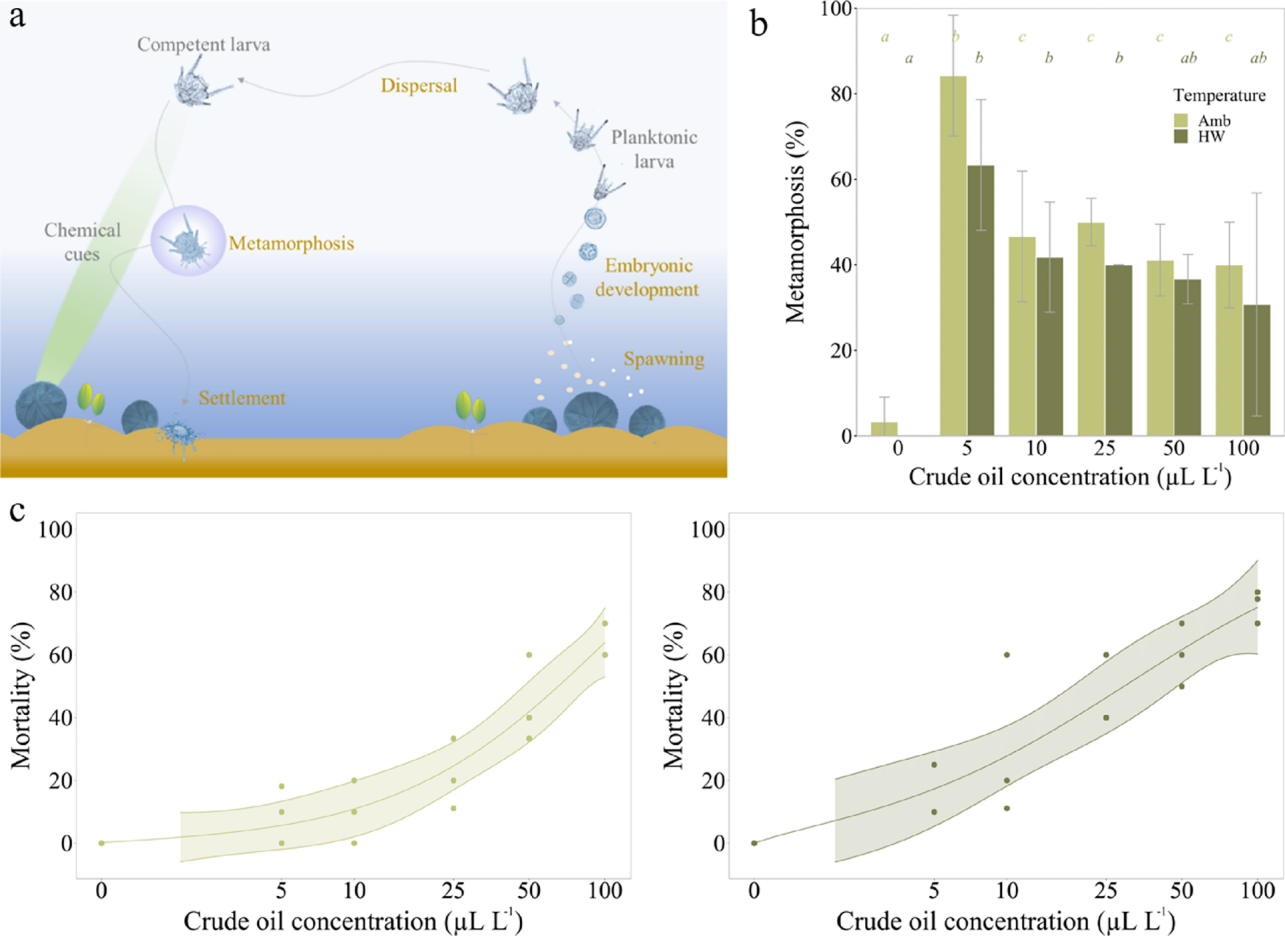
Survival and metamorphosis of competent sand dollar (*Dendraster excentricus*) larvae after 3 days of exposure to crude oil. (a) Schematic of the normal life cycle. (b) Effect of crude oil on metamorphosis (Amb = ambient temperature treatment (13 °C), HW = heat wave treatment (18 °C)). Data are presented as means (*n* = 3) with standard deviations. Lowercase italic letters (*a*, *b*, *c*) indicate different statistical groups (*p* < 0.05) and refer to the color-matching temperature treatment. (c) Effect of crude oil on survival at ambient temperature (left panel) and in the heat wave treatment (right panel).

**Figure 4:**
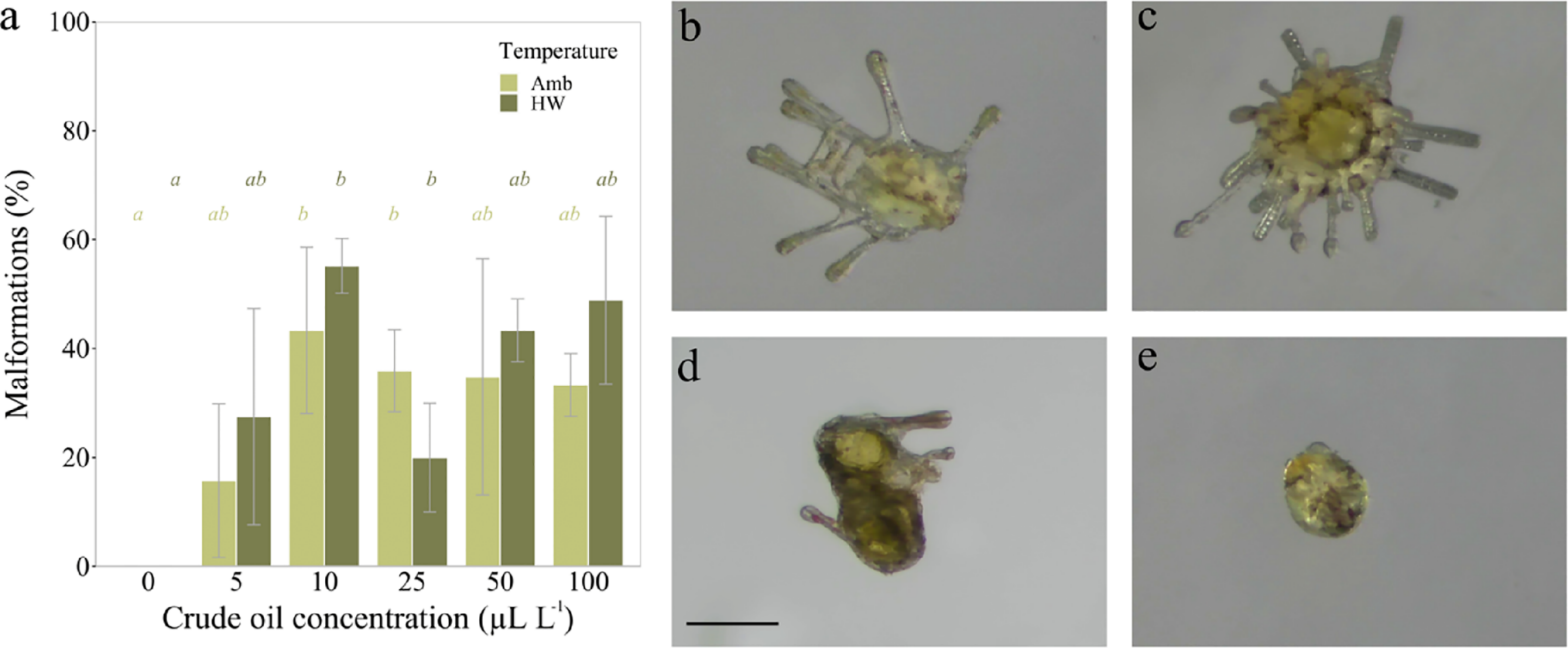
Development of competent sand dollar (*Dendraster excentricus*) larvae after 3 days of exposure to crude oil. (a) Fraction of larvae with malformations (Amb = ambient temperature treatment (13 °C), HW = heat wave treatment (18 °C)). Data are presented as means (*n* = 3) with standard deviations. Lowercase italic letters (*a*, *b*, *c*) indicate different statistical groups (*p* < 0.05) and refer to the color-matching temperature treatment. (b–e) Images of larvae at the end of exposure: (b) competent larva that has not metamorphosed, (c) metamorphosed larva, and (d, e) malformed larvae. Scale bar = 200 μm.

The sand dollar larvae in the control treatments barely showed changes in the studied end points within the 3 days of the experiment (Figures 3 and 4). In contrast, crude oil exposure markedly affected metamorphosis, mortality, and malformations at all studied oil concentrations (Figures 3 and 4). Exposure to crude oil led to a substantial increase in metamorphosed juveniles at all concentrations and at both temperatures (Figure 3). While only 3.3 and 0% of the larvae had metamorphosed in the controls at ambient and increased temperature, respectively, between 30.7 and 84.2% of the oil-exposed larvae had undergone metamorphosis after 72 h (Figure 3). Metamorphosis was highest (84.2%) at the lowest crude oil concentration of 5 μL L^–1^ at ambient temperature (Figure 3). From 10–100 μL L^–1^, there was a slight but nonsignificant trend of decreasing levels of metamorphosis, especially at increased temperature (Figure 3). Metamorphosis was consistently higher at ambient temperature, and the difference between temperature treatments was almost significant (ANOVA: *F* = 4.69, *p* = 0.05).

While there was no mortality in the two controls, it continuously increased with increasing crude oil concentration, from 9.4 and 15% at 5 μL L^–1^ to 63.3 and 75.9% at 100 μL L^–1^, at ambient and increased temperature, respectively (Figure 3). There was a significant effect of temperature on larval mortality, with consistently higher levels of mortality at increased temperature (ANOVA: df = 1, *F* = 12.19, *p* = 0.002). The LC_50_ value in the heat wave treatment was 31 μL L^–1^ in comparison to 102 μL L^–1^ at ambient temperature.

We found malformations of larvae in all treatment groups exposed to crude oil (Figure 4). These included regression of soft tissues around the rods of the arms, complete regression of arms, and a substantial decrease in size (Figure 4). No larvae in the controls showed signs of malformations. The percentage of larvae with malformations ranged from 15.8 to 43.3% at ambient temperature and from 20 to 55.2% at increased temperature (Figure 4). Malformations were generally higher between 10 and 100 μL of crude oil L^–1^, in comparison to 5 μL L^–1^, except for 25 μL L^–1^ at increased temperature (Figure 4). In all but this treatment group, the level of malformations was higher at increased temperature, although this difference was not significant. There was no interaction between the factors “crude oil concentration” and “temperature” for any of the studied end points (ANOVA: *p* > 0.05).

The concentration of total PAHs in the exposure oil solutions ranged between 4.7 and 53.9 μg L^–1^ (Table S1). Naphthalene, acenaphthylene, fluorene, and phenanthrene were the main PAHs found in the crude oil exposure solution used for the echinoderm larva test (Table S1).

## 4 Discussion

Our results demonstrate that exposure to crude oil triggers metamorphosis in marine invertebrates, indicating that petroleum compounds can act as metamorphic inducers. This discovery is groundbreaking since known metamorphosis-inducing substances are typically chemicals from appropriate substrata, microbial biofilms, or conspecifics (pheromones). This is the first evidence that a pollutant of global concern can have this effect on marine animals.

### 4.1 Crude Oil as an Exogenous Chemical Cue Triggering Metamorphosis

Previous studies have pointed out that certain pollutants (e.g., metals, phenols, and petroleum hydrocarbons) can have an inhibitory effect on metamorphosis in marine invertebrates^52^ and that the settlement of some marine invertebrates is reduced in polluted areas.^53^ However, we found that exposure to raw or burnt crude oil can act as a trigger for metamorphosis in invertebrate larvae. Metamorphic and settlement triggers are diverse and commonly species-specific.^15−20,54−56^ Although there is solid evidence that natural chemical cues are primary inducers for metamorphosis in invertebrate larvae, the identification and chemical characterization of the specific molecules acting as metamorphic triggers are still developing.^12,13^ Identified natural metamorphic inducers include microbial lipidic, polysaccharide, or proteinogenic compounds from biofilms,^13^ degradation products from riboflavin (vitamin B2) such as the lumichrome,^57^ and different metabolites such as purines.^56^

Crude oil contains hundreds of different chemical compounds including organic (e.g., alkanes, cycloalkanes, and polycyclic and heterocyclic aromatic hydrocarbons) and inorganic substances (e.g., sulfides, metals). The concentrations of the individual PAHs in the exposure solutions of burnt oil and soot were low (<20 ng L^–1^) since most PAHs are destroyed after burning. The concentrations of PAHs in the mesocosms decreased from day 1 to day 10 (Figure S1), likely due to bacterial degradation, but metamorphosis in gastropod larvae was consistently induced in all the experiments with burnt oil even at the low concentrations of PAHs found in the experiment with water collected on day 10 (Figure S1). Thus, it is unclear if the measured PAHs were the main triggers of metamorphosis or if other oil compounds present in the exposure water induced this effect. Sulfides (e.g., H_2_S), natural anaerobic degradation products of organic matter, induce metamorphosis and settlement in the polychaete *Capitella* sp.^58^ Sulfides are also present in crude oil, but it is unknown if this toxicant can act as a metamorphic inducer for other species not adapted to live in sediments/habitats rich in sulfides. Some of the chemically characterized natural metamorphic inducers like lumichrome and corallinafuran contain aromatic groups in their molecular structure, which could be mimicked by some aromatic compounds in crude oil. However, since we found crude oil to trigger metamorphosis in larvae from two different phyla, the possibility that certain oil compounds mimic two different natural specific metamorphic inducers seems improbable. This could indicate that the oil induction of the metamorphic pathway is rather unspecific, similar to the effect of organic solvents.^59,60^ Pennington and Hadfield (1989)^61^ found 10 organic solvents to induce metamorphosis of competent larvae of the nudibranch *Phestilla sibogae*. Based on the diversity of solvents acting as artificial inducers, they concluded that specific functional groups of the solvent molecules were not required. Our two experiments taken together show that the inducing compounds were present in raw as well as burnt crude oil but not in the soot. We hope that our findings stimulate future research to chemically identify if and what specific petroleum compounds are the primary metamorphic inducers for invertebrate larvae. This is particularly relevant to evaluate if other petroleum products (e.g., gasoline and other light distillates) could have the same harmful sublethal effect on marine invertebrates.

### 4.2 Influence of Environmental Stressors on Metamorphosis: Oil-Triggered Metamorphosis as a Stress Response in Marine Invertebrate Larvae?

Metamorphosis of benthic invertebrate larvae is particularly sensitive to environmental changes/stressors, including pollution.^52^ Besides specific chemical cues, stressful environmental conditions can induce metamorphosis in some invertebrates.^28,31,62,63^ There is also the possibility that the presence of oil causes a stress response that triggers changes in gene expression and molecular processes in the metamorphosis pathway in marine larvae.

Food limitation is an environmental stressor that stimulates metamorphosis of the marine gastropod *Crepidula fornicata*.^28^ We did not find any effect of food availability on metamorphosis in our studied gastropod larvae, which indicates that the observed metamorphosis in the experimental oil treatments was not caused by food limitation. Thermal stress, in the form of a sudden increase in temperature (heat shocks), also induces metamorphosis in some invertebrate species, such as the hydroid *Hydractinia echinata*, the tunicate *Ciona intestinalis*, and the gastropod *C. fornicata*.^31,62^ In contrast, we found no induction of metamorphosis by increased temperature in our studied echinoderm species but rather a consistent pattern of lower metamorphosis in comparison to that of the ambient temperature. This may be a taxon-specific difference or related to the fact that the increase in temperature (5 °C) was lower than those causing metamorphosis in other invertebrate larvae,^31,62^ and our exposure temperature was within the thermal tolerance limit of *D. excentricus*.^64^ Although oil-triggered metamorphosis was not significantly affected by the temperature, a higher temperature increased the mortality caused by crude oil. Synergistic effects of combined exposure to pollutants and increased temperature have been reported for many marine invertebrates.^65^ Here, this could be the result of the higher bioavailability of toxic dissolved petroleum compounds since the solubility of PAHs increases with temperature.^66^ Furthermore, higher temperatures increase the metabolic rates of poikilothermic organisms, which results in a higher energy expenditure.^67^ This may have led to less energy being available for stress response mechanisms.

Any of these factors signal unfavorable conditions in the water column, promoting larval metamorphosis to change habitats and thereby increase the survival probability. Similarly, toxicants like petroleum hydrocarbons can be sensed by planktonic organisms such as copepods, which swim away to avoid petroleum hydrocarbon patches.^68^ From an evolutionary point of view, we hypothesize that the metamorphosis response of competent larvae to the presence of crude oil in the water column may be a strategy to change the habitat. As our results show, crude oil exposure has detrimental effects on the development and survival of larvae. Thus, moving from the polluted water column to the benthos could increase survival probability, albeit with the trade-off of nonsubstrate selection.

### 4.3 Influence of Bacteria on Metamorphosis

A growing body of literature shows that specific bacterial cues can stimulate larval metamorphosis and settlement in different invertebrate taxa.^12,15,54,69−73^ The characteristics of microbial biofilms seem to have a decisive role in the metamorphosis of some species,^12,54,69,74^ but the actual metamorphosis-signaling cues associated with biofilm communities remain largely unknown.^54,71^ Bacterial compounds stimulating metamorphosis are multiple and diverse, including biofilm surface-bound compounds such as protein-lipopolysaccharides, and stimulatory proteins injected into the larvae by certain bacteria.^70−76^ Among the different marine bacteria that can induce the metamorphosis of larvae, species of *Pseudoalteromonas* (γ-proteobacterium) has been shown to produce metamorphic cues for several species.^15,54,71^ Interestingly, whereas some marine bacteria are negatively affected by crude oil (e.g., SAR11), the growth of *Pseudoalteromonas* spp. is stimulated by oil, becoming dominant in the microbial community of oil-polluted water.^77,78^ In this study, competent gastropod larvae were exposed to natural microbial communities from the water collected in the mesocosms; thus, the exposure media contained bacteria. In the case of the sand dollar larvae, the natural seawater was filtered by 1 μm, which can reduce but not completely avoid the presence of planktonic bacteria in the exposure solutions. Therefore, there is a possibility that oil stimulated certain bacteria related to metamorphosis activation, causing the observed effect indirectly. Future research on the interactions among petrogenic compounds, bacteria, and metamorphosis is needed to assess the influence of oil on bacterially induced metamorphosis and to evaluate direct or indirect mechanisms of oil-induced metamorphosis.

### 4.4 Is Crude Oil an Agonist “Endocrine-Disrupting Chemical” (EDC) in Marine Invertebrates?

Physiological and molecular mechanisms underlying metamorphosis are well-known for amphibians and insects^79,80^ but not fully understood for marine invertebrates.^81−83^ Endocrine systems in marine invertebrates are primarily composed of neuroendocrine components, except for crustaceans, which present endocrine glands.^84^ Thyroid hormone receptors and adrenergic receptors were found to play a role in the induction/regulation of metamorphosis in various marine invertebrate larvae, and a number of inducing and inhibiting compounds have been identified.^85−90^ Neurotransmitters are mediators between exogenous metamorphic cues detected by sensory organs (e.g., serotonergic cells in the apical organ of *Aplysia* gastropod veliger) and subsequent metamorphic changes in invertebrate larvae.^86,91^ The potential of chemical pollutants to interfere with endocrine systems was raised several decades ago,^92^ and EDCs have been identified mostly for vertebrates.^84^ Crude oil compounds like PAHs and their alkylated analogues can cause steroidogenic alteration in vitro human cells, acting as potential endocrine disruptors.^93^ However, the underlying molecular mechanisms of metamorphosis activation and the role of hormones or neurohormones in marine invertebrates are still poorly understood. Our findings suggest that crude oil may directly activate the chemical messengers involved in signal transduction for metamorphosis or indirectly enhance bacterially induced metamorphosis. The first case implies that certain petrogenic compounds could act as agonist EDCs in marine invertebrates, a hypothesis that requires further work at the molecular level to be validated.

### 4.5 Ecological Implications

The application of remote sensing to investigate oil pollution has demonstrated the concerning current level of global chronic oiling in the oceans, particularly in coastal areas.^38,94^ Based on our results, the concentrations of crude oil that can induce metamorphosis in invertebrate larvae can be found in coastal areas exposed to chronic pollution and after accidental oil spills.^95−99^ For instance, concentrations of up to 241 μg L^–1^ for total petroleum hydrocarbons were detected in surface waters at Shandong Peninsula (China)^100^ while concentrations of total petroleum hydrocarbons in the highly industrialized Gulf of Trieste, Italy reached 43.2 μg L^–1^.^101^ In both cases, this was mainly attributed to oil pollution from shipping. The observed oil exposure concentration causing the highest metamorphosis induction in the echinoderm larvae experiment (5 μL L^–1^, ∼4.2 ppm) is also environmentally relevant considering the legal upper limits for oil discharges from shipping effluents (15 ppm) and the oil extraction industry (30 ppm for “produced water”).^102−104^ In our studied echinoderm larvae, a total PAH concentration of 4.68 μg L^–1^ was detected in the exposure solution of 5 μL of L^–1^ (Table S1). Although it is not clear whether PAHs are the primary drivers of metamorphic induction, the exposure PAH concentrations are also in the range of concentrations found in the water column in coastal areas.^105−108^ Therefore, there is a high risk of marine invertebrate metamorphic induction by oil compounds in coastal areas that are exposed to oil spills or chronic oiling.

**Figure 5:**
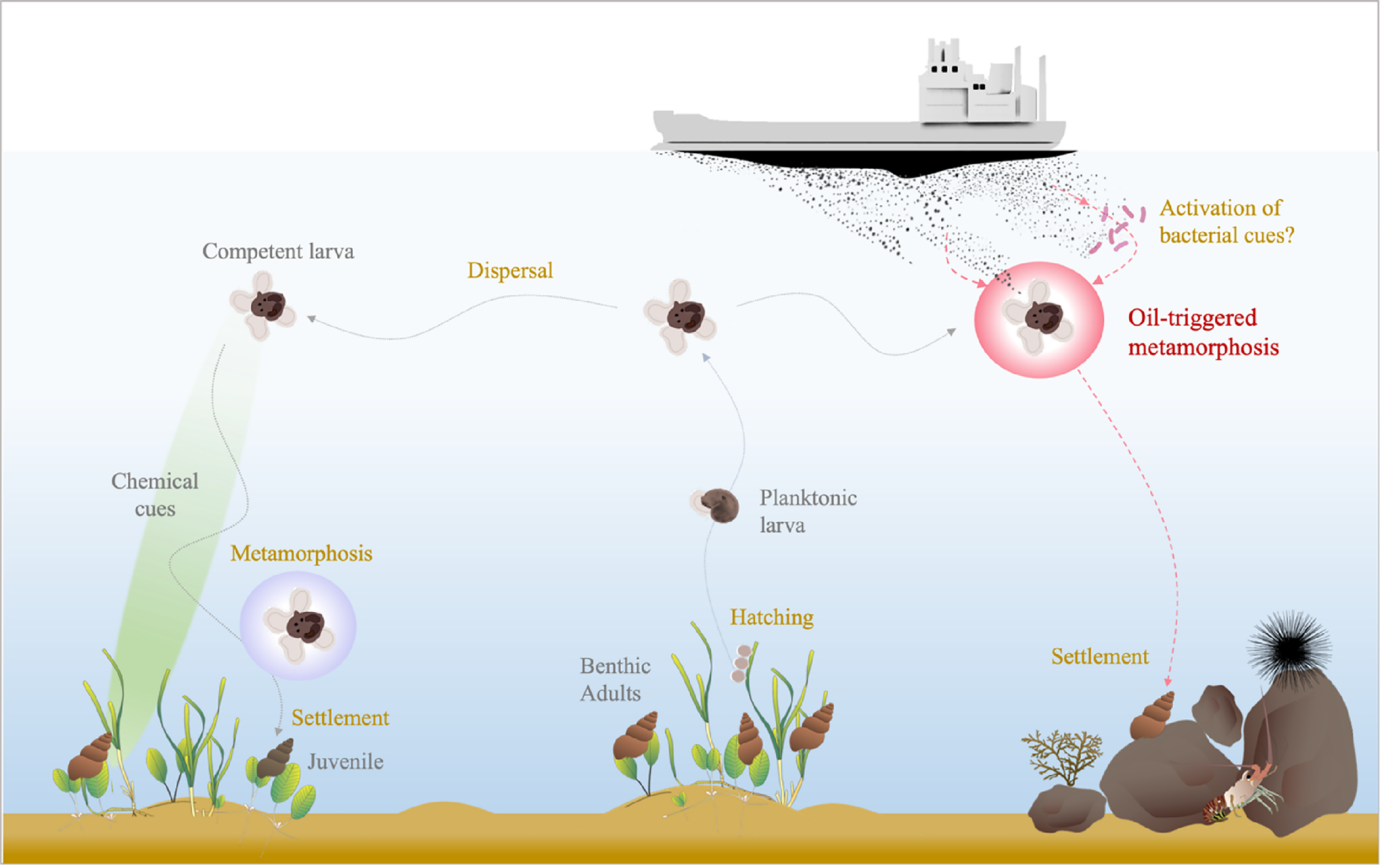
Schematic of the normal life cycle of gastropods with planktonic larvae (left side) and the potential impact of oil pollution on this process (right side). After hatching, larvae swim in the plankton and disperse. Once they reach competence, they can react to chemical cues indicating suitable settlement substrates, undergo metamorphosis, and settle. Oil pollution can trigger metamorphosis and settlement in the absence of appropriate cues and result in settlement in unsuitable habitats.

As mentioned before, marine invertebrate larvae can detect specific chemical cues that indicate favorable conditions for settlement, “metamorphic triggers” (Figure 5). In the absence of metamorphic triggers, competent larvae can delay metamorphosis and continue dispersing; an adaptive strategy that increases the likelihood of settlement in habitats suitable for survival and reproduction.^33,44,109^ This has been demonstrated for the studied echinoid species *D. excentricus*, which can delay settlement until being exposed to chemical cues from conspecific adults. This increases survival probability since the reworking of sediments by adults reduces the occurrence of predators of larvae and juveniles.^34,110^ Delay of metamorphosis has also been observed in gastropods,^33^ and depending on the species, gastropod larvae can detect cues related to appropriate food for juveniles, their algal substrate, and/or conspecific cues (Figure 5). In the presence of oil pollution, oil-induced metamorphosis short-circuits this adaptive metamorphic delay, reducing dispersal and preventing the selection of a suitable habitat for settlement, with potentially severe consequences for the survival of juveniles and recruitment (Figure 5). To determine the potential scale of this effect, more research with different taxa is urgently needed. The ecological consequences of oil-induced metamorphosis are unknown, but it can be surmised to negatively affect the recruitment success of marine invertebrates and consequently marine biodiversity, particularly in coastal ecosystems.

## Supporting Information Available

Figure S1: Concentration and composition of the polyaromatic hydrocarbons detected in the exposure solutions used for the experiments with gastropod larvae. The exposure solutions were collected from the mesocosms (treatments: control, soot, burnt oil) (a) 1, (b) 6, and (c) 10 days after the oil burning; Table S1: PAH concentrations in the exposure solutions (0–50 μL of crude oil L^–1^) used in the experiment with sand dollar *Dendraster excentricus* larvae. All 16 US EPA priority PAHs were measured, but only those with values above the detection limit (0.25 μg L^–1^) are shown (PDF)
