# Supplementary material for: Crude Oil and Its Burnt Residues Induce Metamorphosis in Marine Invertebrates
Source: Environ Sci Technol. 2023 Nov 14;57(48):19304–15. doi: 10.1021/acs.est.3c05194 (PMC10702519; doi:10.1021/acs.est.3c05194)
Supplement: Supplementary file 1 — es3c05194_si_001.pdf [file es3c05194_si_001.pdf]

Supporting information

Manuscript: **Crude oil and its burnt residues induce metamorphosis in marine invertebrates**

Authors: Rodrigo Almeda, Sinja Rist, Anette Maria Christensen, Eleftheria Antoniou, Constantine Parinos, Mikael Olsson, Craig Young

FIGURE S1. Concentration and composition of the polyaromatic hydrocarbons detected in the exposure solutions used for the experiments with gastropod larvae. The exposure solutions were collected from the mesocosms (treatments: control, soot, burnt oil) one (a), six (b), and ten (c) days after the oil burning.

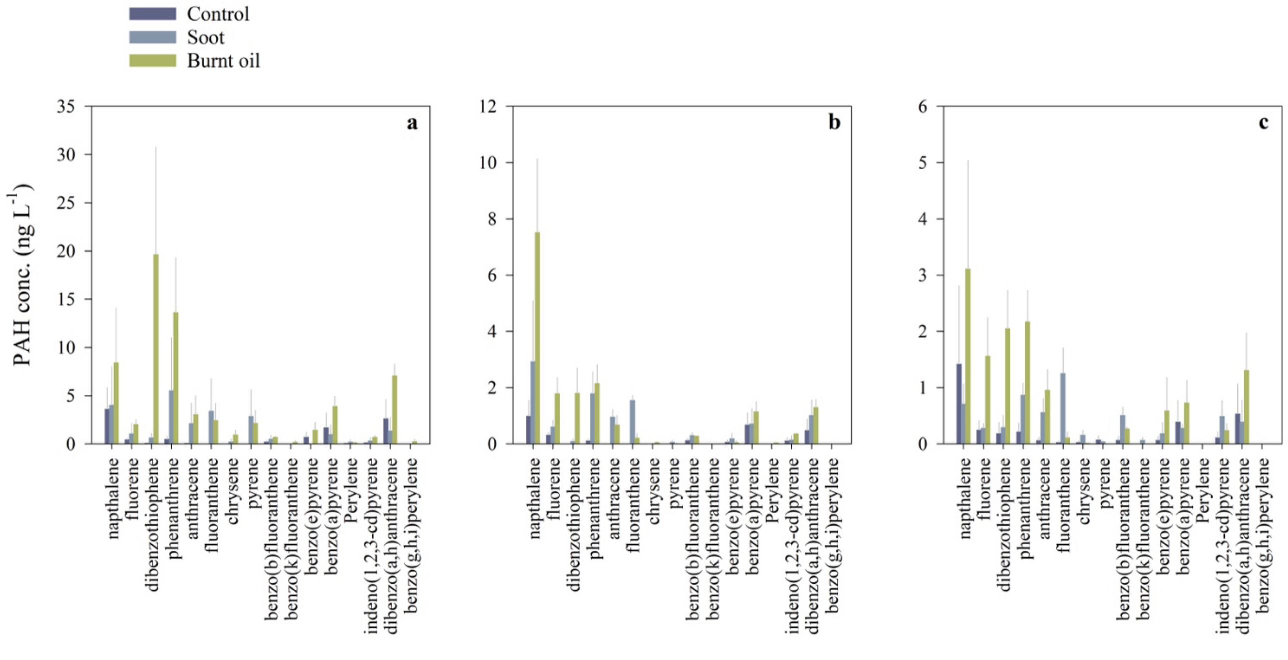

TABLE S1: PAH concentrations in the exposure solutions (0-50 µL of crude oil L^{-1}) used in the experiment with sand dollar *Dendraster excentricus* larvae. All 16 US EPA priority PAHs were measured, but only those with values above the detection limit (0.25 µg L^{-1}) are shown.

	**PAH above detection limit (µg L^{-1})**			
Crude oil conc. (µL L^{-1})	Naphthalene	Acenaphthylene	Fluorene	Phenanthrene
0	0	0	0	0
5	2.7 ± 0.23	0	0.69 ± 0.04	1.29 ± 0.14
10	5.8 ± 0.91	0	1.52 ± 0.21	2.57 ± 0.33
25	12.7 ± 1.63	0.09 ± 0.15	3.22 ± 0.46	4.63 ± 0.48
50	35.5 ± 5.77	0.69 ± 0.14	7.95 ± 1.72	9.77 ± 1.79
